# Diagnostic value of routine endoscopic studies in staging locally advanced cervical cancer: a multicenter experience from a low and middle-income country

**DOI:** 10.3332/ecancer.2026.2139

**Published:** 2026-06-04

**Authors:** Jorge Hoegl, Andreina Fernandes, Fernando Hidalgo, Sunangela Escalona, Anthony López, Yetsy Muñoz, Paola Morillo, Mary Carmen Hidalgo, Luisa López, María Briceño, María Mercedes Pérez

**Affiliations:** 1 Cátedra de Ginecología, Escuela de Medicina José María Vargas, Facultad de Medicina, Universidad Central de Venezuela, Caracas 1010, Venezuela; 2 Laboratorio de Genética Molecular, Instituto de Oncología y Hematología, MPPS, Caracas 1050, Venezuela; 3 Hospital Oncológico Miguel Pérez Carreño, Servicio de Ginecología Oncológica, Valencia 2001, Venezuela; 4 Centro Médico de Oncología, Unidad de Ginecología Oncológica, Barquisimeto 3001, Venezuela; 5 Policlínica de Especialidades Punto Fijo, Unidad de Ginecología Oncológica, Punto Fijo 4102, Venezuela; 6 Departamento de Ginecología y Obstetricia-Ginecología, Hospital General del Este “Dr. Domingo Luciani”, Caracas 1071, Venezuela

**Keywords:** cervical cancer, locally advanced, cystoscopy, rectosigmoidoscopy, magnetic resonance imaging

## Abstract

**Objective:**

To evaluate the diagnostic value of routine cystoscopy and rectosigmoidoscopy in staging locally advanced cervical cancer (LACC) in a low - middle - income country (LMIC) setting.

**Material and methods:**

A multicenter, retrospective cohort study of 233 patients with LACC (International Federation of Gynecology and Obstetrics 2018 stages IB3–IVB) in Venezuela. All patients underwent clinical examination, magnetic resonance imaging (MRI) and routine endoscopy. The primary outcome was the confirmation of mucosal invasion by endoscopy in patients without prior clinical or radiological suspicion.

**Results:**

Endoscopically confirmed bladder and rectal mucosal invasion occurred in 6.0% and 3.0% of patients, respectively. In 95% of all positive cases, suspicion was already present on MRI or clinical symptoms. MRI demonstrated high negative predictive values for bladder (95.9%, confidence interval (CI) 95% 92.5–98.1) and rectal (97.4% CI 95% 92.2–97.8) invasion. Notably, no treatment plan was modified solely based on endoscopic findings. Routine endoscopy was negative in 94% of cystoscopies and 97% of rectosigmoidoscopies.

**Conclusion:**

Routine endoscopic staging in LACC showed a very low diagnostic yield and no impact on therapeutic decisions in this LMIC setting. A selective, imaging–guided approach is safe, resource–efficient and aligns with international guidelines, advocating for a change in local protocols. These findings strongly support discontinuing routine endoscopy in LMIC staging protocols.

## Introduction

Cervical cancer remains a global health challenge. It is currently the fourth most common gynecological cancer in women, with over 600,000 new cases and approximately 340,000 deaths each year. The burden is disproportionately higher in low- and middle-income countries (LMIC) where between 55% and 80% of cases are diagnosed at locally advanced stages, resulting in poorer oncological outcomes and limited access to effective prevention and screening programs [1]. In Venezuela, 3,965 new cases are diagnosed annually, with mortality reaching 2,246 cases, resulting in a mortality rate of over 50% [2]. Meanwhile, the proportion of locally advanced cervical cancer (LACC) cases ranges from 70% to 85%, leading to poorer oncological outcomes in terms of disease-free survival and overall survival [3, 4]. This disparity highlights not only barriers to prevention but also limitations in screening strategies and accessible, cost-effective methods for accurate staging [5].

Accurate staging of LACC is critical for prognosis and treatment planning. In 2018, the International Federation of Gynecology and Obstetrics (FIGO) included imaging studies, such as computed tomography (CT), magnetic resonance imaging (MRI) and ultrasound (transvaginal, rectal and abdominal), as important tools in the cervical cancer staging system. It considers invasive procedures like cystoscopy and rectosigmoidoscopy to be optional, reserved for patients with clinical or radiological suspicion of bladder or rectal invasion [6–8]. Despite these recommendations, some countries continue to endorse the routine use of these endoscopic studies in staging workflows, regardless of clinical or imaging findings, resulting in discrepancies with international practice guidelines [6, 7, 9]. These decisions highlight how real-world practice may lag behind international standards, especially in LMIC.

Recent studies have shown that imaging modalities achieve high specificity and negative predictive value (NPV) for detecting bladder and/or rectal involvement, questioning the additional diagnostic value and cost-effectiveness of routine endoscopy [10–13]. However, clinical staging inaccuracies are still common in LMICs, with rates ranging from 20% to 60%. This is partly due to resource constraints and the limited availability of experienced examiners. This has prompted an ongoing debate about whether cystoscopy and rectosigmoidoscopy should be applied routinely or selectively [11, 14].

Due to the differences between guideline recommendations and real-world practice, robust empirical data from LMIC are needed to determine if routine endoscopic assessment provides additional diagnostic or management value compared to a selective, imaging-guided approach. However, unlike previous reports primarily from high-income countries, there is a paucity of robust, multicenter evidence from Latin America, where the burden of LACC is disproportionately high and staging resources are limited. This study addresses this critical gap by providing one of the largest real–world cohorts from the region, offering data that can directly inform practice and policy in LMIC. This multicenter retrospective study aims to evaluate the usefulness of routine cystoscopy and rectosigmoidoscopy in staging LACC, exploring whether their selective application based on clinical and imaging criteria could represent a more rational and guideline–concordant approach.

## Materials and methods

### Study design and setting

An observational, multicenter, retrospective cohort study across four tertiary centers in Venezuela was conducted on patients diagnosed with LACC between January 2015 and December 2022. The study took place at the Gynecology Departments of Hospital General del Este Domingo Luciani in Caracas and Hospital Oncológico Miguel Pérez Carreño in Valencia, as well as at Policlínica de Especialidades Punto Fijo and Centro Médico de Oncología de Barquisimeto. All centers are referral institutions for gynecologic oncology and serve a predominantly low- to middle-income population. The protocol was reviewed and approved by the Hospital General del Este “Dr. Domingo Luciani” bioethics committee (6/2025).

### Patient selection

Eligible patients were women aged ﻿18 years old, with histologically confirmed, (IB3-IVB) LACC at presentation. Patients were included if they underwent a clinical examination and pelvic imaging studies (MRI) before treatment initiation. Symptoms were systematically recorded from the patient’s clinical record at the initial evaluation. They also underwent both rectosigmoidoscopy and cystoscopy as part of the initial evaluation and staging. Those with early-stage cervical cancer, incomplete clinical or radiological records, missing endoscopic data, non-cervical tumours, use of CT and/or ultrasound or prior pelvic radiation were excluded.

### Data collection

Demographic, clinical and pathological data were collected from institutional databases and patient charts. All data were anonymised before analysis and handled in accordance with the Declaration of Helsinki. Imaging studies were reviewed by board-certified radiologists. Only MRI-based staging was included to ensure diagnostic homogeneity and alignment with contemporary FIGO/ESGO recommendations. Urinary and rectal symptoms were retrospectively extracted from routine clinical records documented at initial patient evaluation. Symptoms were recorded as present or absent based on physician documentation; no standardised symptom severity scale was used and definitions were not formally harmonised across centers. Cystoscopies and rectosigmoidoscopies were performed by urologists or colorectal surgeons, respectively, following local protocols. Endoscopic confirmation of mucosal invasion by cystoscopy or rectosigmoidoscopy with biopsy was considered the pathological reference standard. Clinical symptoms and imaging findings were evaluated as predictive and triage variables rather than independent diagnostic tests. All data were entered into an Excel database and patients who did not meet the inclusion criteria were excluded. Disease staging followed the 2018 FIGO classification.

### Outcomes

The primary outcome was to evaluate the diagnostic yield of routine cystoscopy and rectosigmoidoscopy, defined as the proportion of procedures that confirmed mucosal invasion in patients without prior clinical or radiological suspicion.

### Statistical analysis

After performing data quality control and coding, a matrix was created to transfer the dataset into SPSS Statistical Software, version 27. Quantitative variables were expressed as ranges and means ± standard deviation. Qualitative variables were described using frequencies and percentages. Hypotheses were developed based on specific objectives, and Fisher’s exact test was chosen for dichotomous variables. A *p*-value less than 0.05 was considered statistically significant with a 95% confidence interval (CI). The strength of the association between dichotomous variables was evaluated using the Phi correlation coefficient (φ). Values range from −1 to +1, with values near 0 indicating no association and values near −1 or +1 indicating stronger associations. For reference, φ values of 0.10–0.30 are deemed low, 0.30–0.50 moderate and greater than 0.50 strong. Only patients staged with pelvic MRI were included in the final analysis to ensure diagnostic homogeneity and to align with current FIGO and ESGO recommendations regarding local staging. Given the small number of mucosal invasion events, Fisher’s exact test was used for categorical comparisons and relative risks (RRs) were calculated to express the magnitude of association.

RR was computed as the ratio of the confirmation rate in the group with the predictor factor to that in the group without it. An RR greater than 1 indicates increased risk, an RR of 1 indicates no effect and an RR less than 1 indicates decreased risk. The diagnostic accuracy of endoscopic tests was assessed using sensitivity, specificity, positive predictive value (PPV) and NPV, with clinical and imaging findings serving as the reference standard.

## Results

**Figure 1.**
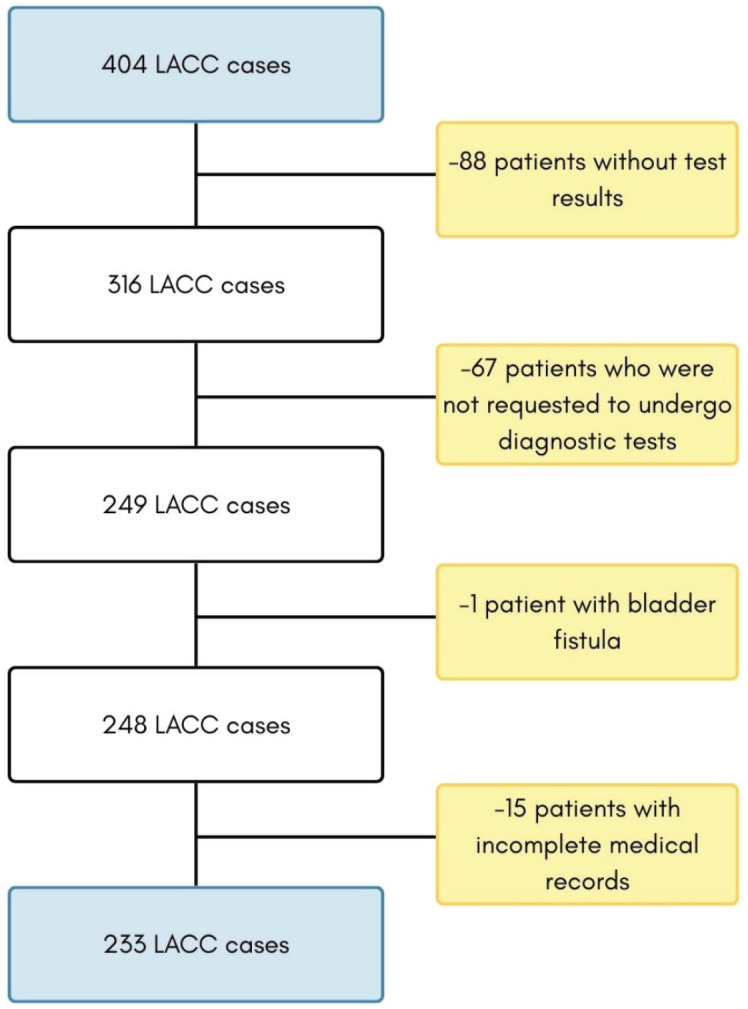
Flowchart of cervical cancer case exclusion, according to inclusion criteria. LACC: locally advanced cervical cancer.

Of the total number of cases reviewed between 2015 and 2022, 404 cases of patients diagnosed with LACC were selected. Figure 1 shows the flowchart of exclusion of patients who did not meet the inclusion criteria, resulting in a final sample of 233 patients with LACC. Of the 233 patients evaluated, routine endoscopy demonstrated a low diagnostic yield. The vast majority of procedures (94% of cystoscopies and 97% of rectosigmoidoscopies) revealed no mucosal invasion. No patient was upstaged to FIGO stage IVA solely based on endoscopic findings. All patients with endoscopically confirmed mucosal invasion had pre-existing clinical or imaging suspicion, and endoscopic results did not independently alter treatment modality, radiation field design, dose escalation or brachytherapy approach*.*

Table 1 summarises the characteristics of the selected study population. The median age of the patients was 49.9 ± 12.2 years, with a median age of 49 years (95% CI 48.3–51.4). The youngest patient was 25 years old, and the oldest was 84 years old. The most representative age range was between 45 and 63 years old, accounting for 48.1% (112/233) of patients. The most frequent stage was IIB (45.9%), and squamous cell carcinoma was the most prevalent histological type (92.7%). Urinary symptoms were reported in 7.3% (17/233) of patients, while rectal symptoms were reported in 5.6% (13/233). Regarding imaging studies, 5.2% (12/233) of patients were diagnosed with bladder invasion and 2.1% (5/233) with rectal invasion. Finally, 6% (14/233) and 3% (7/233) of patients were diagnosed with tumour invasion by cystoscopy and rectosigmoidoscopy, respectively

**Figure 2.**
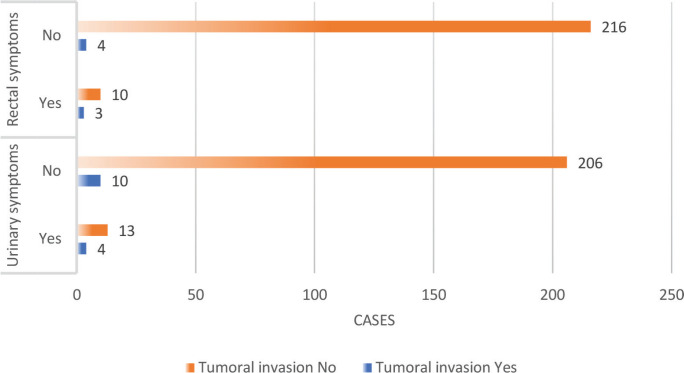
Patients with LACC and urinary and rectal symptoms, diagnosed with tumour invasion by cystoscopy and rectosigmoidoscopy.

Among the 17 patients with urinary symptoms, 23.5% (4/17) were diagnosed with tumour invasion by cystoscopy and biopsy. In contrast, only 4.6% (10/216) of the 216 patients without urinary symptoms were diagnosed with tumour invasion by cystoscopy and biopsy. Among the 13 patients with rectal symptoms, 23.1% (3/13) were diagnosed with tumour invasion by rectosigmoidoscopy and biopsy. In comparison, only 1.8% (4/220) of the 220 patients without rectal symptoms were diagnosed with tumour invasion by rectosigmoidoscopy and biopsy (Figure 2).

**Figure 3.**
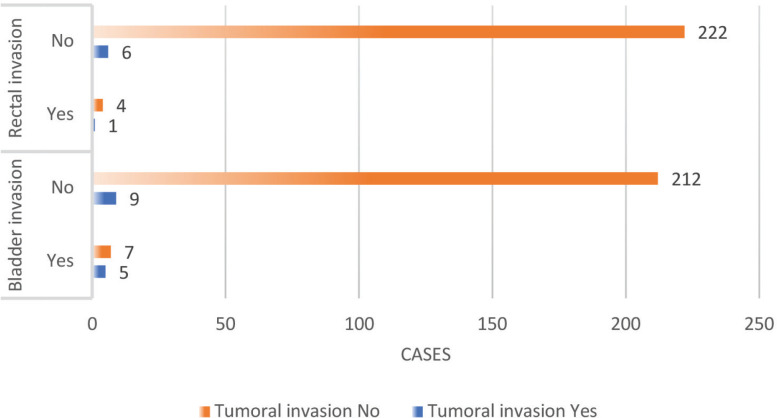
Patients with LACC and bladder and rectal invasion on imaging, diagnosed with tumour invasion by cystoscopy and rectosigmoidoscopy.

Of the 12 patients with bladder invasion on imaging, 41.7% (5/12) were diagnosed with tumour invasion by cystoscopy and biopsy. Of the 221 patients without bladder invasion, 4.2% (9/221) were diagnosed with tumour invasion by cystoscopy and biopsy. Of the five patients with rectal invasion on imaging, 20% (1/5) were diagnosed with tumour invasion by rectosigmoidoscopy and biopsy. Of the 228 patients without rectal invasion on imaging, only 2.6% (6/228) were diagnosed with tumour invasion by rectosigmoidoscopy and biopsy (Figure 3).

Hypothesis tests were performed to evaluate the association between clinical, radiological and histological variables. The results of Fisher’s exact tests and Phi correlation coefficients (rφ) are presented in Table 2. Patients with urinary symptoms were 5.1 times more likely to be diagnosed with tumour invasion than patients without these symptoms (23.5% versus 4.6%), while patients with rectal symptoms were 12.8 times more likely to have confirmed tumour invasion than patients without symptoms (23.1% versus 1.8%). A low correlation coefficient was found for both variables.

For patients with an imaging diagnosis of bladder invasion, there is an almost 10-fold higher probability that cystoscopy will confirm invasion (41.7% versus 4.1%), with a moderate Phi coefficient. A positive imaging finding result is associated with a 7.7 times higher risk of confirmation by rectosigmoidoscopy (20% versus 2.6%), with a low correlation coefficient. Given the small sample size, these results should be interpreted with caution.

Table 3 shows the diagnostic performance of clinical symptoms and imaging findings for predicting bladder and rectal invasion confirmed by endoscopy. Urinary symptoms demonstrated a sensitivity of 28.6% (95% CI 8.4–58.1) and a specificity of 94.1% (95% CI 90–96.8) for detecting bladder invasion, with a PPV of 22.5% (95% CI 6.8–49.9) and an NPV of 95.1% (95% CI 91.7–97.7). Rectal symptoms showed a sensitivity of 42.9% (95% CI 9.9–81.6) and a specificity of 95.6% (95% CI 95.4–99.5) for detecting rectal invasion, with a PPV of 23.1% (95% CI 5.0–53.8) and a NPV of 98.2% (95% CI 96.7–99.9). When imaging findings were compared against endoscopic confirmation, sensitivity was low (35.7% (95% CI 12.8–64.9) for bladder and 14.3% (95% CI 0.4–57.9) for rectal invasion), although the NPV remained high (above 95% for both). In this cohort, the vast majority of routine endoscopic procedures (94% of cystoscopies and 97% of rectosigmoidoscopies) did not reveal tumour invasion. No treatment plan was altered solely on the basis of endoscopic findings.

## Discussion

This multicenter retrospective study provides one of the largest cohorts and compelling evidence from a real-world LMIC setting against the routine use of cystoscopy and rectosigmoidoscopy in staging LACC. We demonstrated a strikingly low prevalence of endoscopically confirmed bladder (6.0%) and rectal (3.0%) invasion. Crucially, in the vast majority of these cases, the suspicion of invasion was already raised by pre–existing clinical symptoms or imaging findings. Rather than serving as definitive diagnostic tests, clinical symptoms and imaging findings function as effective triage tools to identify patients who may benefit from confirmatory endoscopy. In this context, the high NPV of MRI is particularly relevant, as it allows for safe exclusion of mucosal invasion and supports the omission of routine invasive procedures. Most importantly, not a single treatment was altered based on the endoscopic findings alone, as all patients with confirmed invasion had their disease management correctly guided by imaging and clinical staging, culminating in definitive chemoradiation. This finding directly challenges the utility of routine endoscopy and strongly supports a selective, imaging–guided approach, as recommended by FIGO and ESGO guidelines [7, 15, 16].

Our results are consistent with evidence from diverse international settings. Anfinan *et al* [11] documented that despite local routine use of endoscopy, MRI achieved a near 100% NPV for ruling out bladder and rectal invasion, concluding that endoscopy could be omitted in the absence of clinical or radiological suspicion. Similarly, Sapienza *et al* [10] found high NPVs for imaging (98% and 100% for bladder and rectal involvement, respectively), safely allowing the omission of routine endoscopies. Our data corroborates these findings, showing a low prevalence of endoscopy–confirmed bladder (6.0%) and rectal (3.0%) invasion. The small number of rectal invasion events resulted in wide CIs and unstable effect estimates; therefore, these findings should be interpreted with caution. Furthermore, the European multicenter EMBRACE study reported that cystoscopy and rectosigmoidoscopy were performed only in 41% and 12% of cases, respectively, at the physician’s discretion. In that cohort, MRI demonstrated a sensitivity of 96%, specificity of 93% and a NPV of 99.8% for bladder invasion, confirming that advanced imaging largely replaces the need for routine endoscopy [17]. Although our study originates from a resource–limited context, the conclusions point in the same direction: the prevalence of confirmed invasion was low, imaging showed high NPVs (≥95%) and endoscopy did not alter the therapeutic plan.

Considering this data, the predictive value of symptoms and imaging becomes clear. The presence of urinary or rectal symptoms significantly increased the RR of confirmed invasion, yet their low prevalence means they lack utility as distinct screening tools. More critically, the high NPV of imaging (>95% for both bladder and rectum) is the cornerstone of the selective strategy. Furthermore, this means that a negative imaging result reliably excludes invasion with a degree of certainty that makes routine endoscopy redundant. While the PPV of imaging was moderate, reflecting the challenge of distinguishing tumour adhesion from true mucosal invasion, this does not justify subjecting all patients to endoscopy. Rather, it precisely defines the select patient cohort – those with positive or equivocal imaging findings who would benefit from a confirmatory endoscopic procedure. Bonatti *et al* [18] recently provided multicenter evidence supporting a selective MRI-guided approach to staging. In their 214-patient cohort, MRI-defined bladder wall invasion independently predicted recurrence and cancer-specific mortality, whereas cystoscopy-defined mucosal infiltration added no prognostic value. These findings, derived entirely from MRI-based assessments, reinforce these results and confirm that MRI accurately identifies clinically significant bladder involvement, rendering routine cystoscopy unnecessary when imaging is negative.

Beyond the lack of diagnostic yield, avoiding these unnecessary procedures translates to reduced costs, minimised procedure risks and most critically, accelerated initiation of definitive chemoradiation [19, 20]. In cervical cancer, where treatment delays adversely impact survival, streamlining the staging process by omitting low–yield invasive tests is not merely an efficiency gain but a crucial step toward improving oncological outcomes [21, 22]. The interpretation of these findings must be considered in the context of the study’s limitations. The retrospective design inherits the potential for selection and information bias. This retrospective design carries an inherent risk of verification bias, as endoscopic confirmation was not independent of prior clinical and imaging assessment. Furthermore, the small number of rectal invasion events limits the precision of our statistical estimates for this specific outcome. However, the strength of this study lies in its sample size, with 233 patients across four referral centers. This series represents one of the largest Latin American cohorts evaluating the role of routine endoscopy in LACC.

## Conclusion

Our study provides robust evidence from an LMIC setting that routine cystoscopy and rectosigmoidoscopy should no longer be performed systematically in the staging of LACC. These results support the adoption of selective, MRI-guided endoscopic staging protocols, especially in resource-constrained settings where diagnostic rationalisation can directly improve access and treatment timeliness.

## List of abbreviations

CI, Confidence interval; CT, Computed tomography; ESGO, European Society of Gynecological Oncology; FIGO, International Federation of Gynecology and Obstetrics; LACC, Locally advanced cervical cancer; LMIC, Low- and middle-income countries; MRI, Magnetic resonance imaging; NPV, Negative predictive value; PPV, Positive predictive value; RR, Relative risk.

## Conflicts of interest

The authors declare no conflicts of interest.

## Funding

This research received no specific grant from any funding agency in the public, commercial, or not-for-profit sectors.

## Author contributions

**JH:** Conceptualisation, data collection and curation, data analysis, visualisation, writing the original draft, reviewing and editing the manuscript. **AF:** Conceptualisation, data curation and analysis, reviewing and editing the manuscript. **FH, SE, AL, YM, PM, MCH, LL, MB, MMP**: Data collection and curation, reviewing the manuscript.

## Figures and Tables

**Table 1.** General characteristics of patients with LACC.

	N (233)	%
Stages		
IB3	4	1.7
IIA	10	4.3
IIB	107	45.9
IIIA	1	0.4
IIIB	85	36.5
IIIC	12	5.2
IVA	10	4.3
IVB	4	1.7
Histological types		
Squamous cell carcinoma	216	92.7
Adenocarcinoma	16	6.9
Adenosquamous	1	0.4
Urinary symptoms		
Yes	17	7.3
No	216	92.7
Bladder infiltration (by imaging)		
Yes	12	5.2
No	221	94.8
Bladder infiltration (by cystoscopy)		
Yes	14	6
No	219	94
Rectal symptoms		
Yes	13	5.6
No	220	94.4
Rectal infiltration (by imaging)		
Yes	5	2.1
No	228	97.9
Rectal infiltration (by rectosigmoidoscopy)		
Yes	7	3
No	226	97

**Table 2.** Results of hypothesis tests and measures of association.

	n	Tumoural invasion* n (%)	p value	Phi coefficient (rφ)	RR	95% CI
Urinary symptoms						
Yes	17	4 (23.5)	0.012	0.202	5.1	1.74–14.97
No	216	10 (4.6)				
Rectal symptoms						
Yes	13	3 (23.1)	0.004	0.286	12.8	3.33–49.24
No	220	4 (1.8)				
Bladder invasion (by imaging)						
Yes	12	5 (41.7)	0.001	0.350	9.9	3.60–27.24
No	221	9 (4.1)				
Rectal invasion (by imaging)						
Yes	5	1 (20.0)	0.014	0.147	7.7	1.04–56.77
No	228	6 (2.6)				
*Diagnosed by cystoscopy or rectosigmoidoscopy. RR: relative risk						

**Table 3.** Sensitivity and specificity analysis of clinical symptoms and imaging with respect to cystoscopy and rectosigmoidoscopy.

	S (%) (95% CI)	Sp (%) (95% CI)	PPV (%) (95% CI)	NPV (%) (95% CI)
Urinary symptoms vs cystoscopy	28.6 8.4–58.1	94.1 90–96.8	22.5 6.8–49.9	95.1 91.7–97.7
Rectal symptoms versus rectosigmoidoscopy	42.9 9.9–81.6	95.6 95.4–99.5	23.1 5.0–53.8	98.2 96.7–99.9
Imaging versus cystoscopy	35.7 12.8–64.9	96.8 93.6–98.7	41.7 15.2–72.3	95.9 92.5–98.1
Imaging versus rectosigmoidoscopy	14.3 0.4–57.9	98.2 94.5–99.0	20.0 0.5–71.6	97.4 92.2–97.8
**S: sensitivity; SP: specificity; PPV: positive predictive value; NPV: negative predictive value**				
